# Gel-based proteomics in plants: time to move on from the tradition

**DOI:** 10.3389/fpls.2015.00369

**Published:** 2015-05-27

**Authors:** Arun K. Anguraj Vadivel

**Affiliations:** Department of Biology, Western UniversityLondon, ON, Canada

**Keywords:** gel-based proteomics, plant proteomics, gel-free proteomics, mass spectrometry, technical fusion

In more than 20 years of the proteomics era, considerable technical developments and scientific discoveries have contributed to the advancement of this field of research. Gel-based proteomics has been popular for studying the proteomic changes during growth and development of plants as well as for the analysis of responses to different biotic and abiotic stimuli. The most widely used gel-based technique is two dimensional (2D) gel electrophoresis, in which around 2000 protein spots can be clearly visualized and processed prior to identification by mass spectrometry. Although many techniques have been developed to improve the quality and number of spots in a 2D gel, these enhancements are still not good enough to study the entire cellular proteome. It is important to also consider low abundant proteins, many of which could play critical roles in particular biological processes. Considering that thousands of proteins are expressed at a given time and each protein can undergo one or more post-translational modifications—are we able to capture all this information and the entire proteome using gel-based proteomic techniques? Are gel-free alternatives capable of addressing these challenges? The answer is no for both approaches. So, how long are we going to run traditional 1D and 2D gels in our research? Though we have been successful in developing many advanced gel-free proteomic techniques to study the cellular proteome, we still lack advances that enable the examination of each and every protein expressed in a system as well as any post-translational modification (PTM) at a given time. Gel-free and gel-based techniques may complement each other, however there needs to be synergy or a “technical fusion” in which researchers can find a balance between quick and efficient methods for studying whole cell proteomes in plants.

## Proteins and proteomics

Many plants contain more genes than the human genome, which we consider one of the most complex organisms. However, the number of genes cannot be easily correlated with the number of functional proteins since an organism has multiple layers of gene regulation. Gene expression can be regulated during transcription, splicing, translation and post-translational maturation of the protein. Several physical factors such as tissue types, developmental stages, environmental stimuli and stresses also affect gene expression in plants. In addition, due to the number of ploidy levels in some plants, and the presence of several protein isoforms, studying a large plant proteome can be more complex than studying an animal proteome of apparently similar size. Different proteomic tools have been developed in the past few decades, most of which were first used for studying animal proteomes and then later adopted for plants. Chromatography and protein electrophoresis have been ideally used for the separation of proteins whereas mass spectrometry (MS) has been used for protein identification. Different kinds of MS have been developed that use variable ionization sources, fractionation and mass analyzers. Among these, the orbitrap mass analyzer is now the most widely used for high resolution MS. Generally, MS based proteomics is used for protein identification and quantification studies either in combination with gel-based (1D, 2D or 3D) or gel-free techniques and can be used with label free or tag-based techniques (ICAT, iTRAQ etc.). The workflow of MS based proteomics has recently been reviewed by Jorrin-Novo et al. (2015). The review covers all research articles in plant proteomics that have been published in the journal *Proteomics* since 2000. During that period there have been many modifications to proteomic methods used to obtain high protein coverage and improve the number of proteins identified per sample. This article covers some of the more recent studies in the MS workflow before raising the question of how can we improve the output by merging different techniques i.e., development of different methods in gel-based and gel-free techniques to improve protein separation, digestion and recovery of peptides, and MS or MS/MS data analysis.

## Gel-based proteomics

The most widely used methods in gel-based proteomics comprise the separation of proteins by sodium dodecyl sulfate polyacrylamide gel electrophoresis (SDS-PAGE). This can be conducted in a single dimension (1D) based on molecular weight or in two dimensions (2D) based on a proteins isoelectric point (using immobilized pH gradient gel strips) and molecular weight (SDS-PAGE). Identification of proteins in a sample follows; separation by SDS-PAGE, *in-gel* digestion with an enzyme and MS. 2D gel electrophoresis is the most popular approach in plant proteomics as it allows separation of proteins by isoelectric point and molecular mass. Over 4000 proteins can be identified using 2D gel electrophoresis combined with MS (Imin et al., 2001). PTM specific stains have been developed for phosphoproteins (e.g., Pro-Q Diamond) and glycoproteins (e.g., Pro-Q Emerald, Dansylhydrazine), both of which have emerged as good techniques to study specific PTMs using 2D gels (Marondedze et al., 2013; Wang et al., 2014). However, 2D gels have limitations in terms of separation resolution of complex proteomes, number of spots and protein recovery; all which can be improved by merging methods developed separately for each step in the gel-based proteomic work flow. However, current 2D separation techniques are still not adequate, thus some recent developments in this area are outlined.

Recently, Colignon et al. (2013) developed a protocol for the three dimensional separation of proteins. 3D separation is an advanced 2D gel separation method and their protocol was developed to addresses co-migration interferences. It uses isoelectric focusing and sample fractionation followed by two consecutive separations by SDS-PAGE, with two different buffer systems to evade co-migration associated drawbacks that can affect protein resolution. In this study, MS/MS analyses from both 2D and 3D gels were compared to validate the protocol. There are previously reported methods available for 3D gels to study particular protein families (Jiang et al., 2009) but the method reported by Colignon et al. (2013) provides a wide range of applications in quantitative profiling of complex proteomes and in the identification of PTMs such as protein phosphorylation and sumoylation. Interestingly, a 3D-western blot is also possible. The authors suggest that by combining this method with a differential gene expression approach, the relative abundance of modified proteins can be studied in two different samples. However, overcoming extraction problems after protein separation still remains i.e., recovery of proteins from the gel.

One of the critical steps is protein digestion with proteases like trypsin, chymotrypsin, etc. There have been a number of methods developed for peptide recovery after digestion. A tube gel digestion has been developed for protein recovery that does not require protein electrophoresis to study membrane proteins (Lu and Zhu, 2005). Takemori et al. (2014) developed a method to improve peptide recovery from polyacrylamide gels in a tube using a disulfide-containing analog of bis-acrylamide called bis-acrylylcystamine (BAC). In this technique, release of peptides from the gel was enhanced by adding tris-(2-carboxyethyl) phosphine (TCEP) instead of DTT for complete dissolution of the BAC-cross-linked acrylamide gel. BAC gels can be used for complex membrane proteins and can improve recovery prior to MS analysis. However, it is difficult to detect less abundant proteins in the samples even when used in conjunction with nanoLC–MS/MS. However, merging this method with 2D gel technology to create a 2D BAC gel would improve protein recovery from *in-gel* digestion of 2D gel spots and improve identifications. Moreover, developing 3D separation techniques with BAC gels with two different buffer systems, as discussed earlier, would give improved separation resolution to obtain more spots in conjunction with peptide recovery. This represents an excellent fusion of techniques if downstream process can be successfully applied.

The next critical step in the MS workflow is MS or MS/MS data analyses in order to accurately match the correct peptide to the spectra. Silva et al. (2014) discussed several ways to improve data visualization and analyses from both MALDI-TOF and ESI-MS approaches. The study also includes suggestions about feature selection or spot detection in gel-based proteomics, which could be helpful for researchers in this field. However, the data visualization methods discussed in this article only work when upstream processes are carefully monitored. The study also demonstrated the effectiveness of statistical and multivariate tools. As discussed before, developing methods for 3D separation of proteins using BAC gels for higher resolution of separation and recovery of peptides in combination with perfect feature selection for data visualization and multivariate analyses tools could be worth considering in the area of gel-based proteomics. It is undeniable that advances in technical development can take research to the next level; however there is always room for further improvements.

## Gel-free proteomics

Some of the limitations in gel-based proteomics can be ignored when employing gel-free proteomics. However, both techniques can still complement each other, and their selection of either is highly dependent on the sample or the question. The most popular gel-free approach among researchers is multi-dimensional protein identification (MudPIT) comprising strong cation-exchange (SCX) fractionation, reversed-phase (RP) chromatography and tandem mass spectrometer (MS/MS) (Link et al., 1999; Washburn et al., 2001). It comprises *in-solution* digestion instead of *in-gel* digestion. The digested peptide mixture is loaded onto chromatography columns which are in line with the MS/MS. At least 2000 proteins can be identified in a sample using the MudPIT approach (Hernandez et al., 2012). Over 12,000 proteins have been identified in different organs of *Arabidopsis* and in maize leaf (Baerenfaller et al., 2008; Facette et al., 2013). Recently, Link and Washburn (2014) established two approaches to yield high quality tandem mass spectra from complex protein solutions. A multidimensional system is considered comprehensive and highly sensitive approach for protein identification in a complex sample.

A recent study compared an automated (online) or manual (offline) format for MudPIT as well as different quantitative MudPIT strategies using label-free and tandem mass tag (TMT) isobaric tagging (Magdeldin et al., 2014). The study concluded that higher sequence coverage and more peptide/protein identifications can be achieved using online MudPIT rather than when employing offline sample fractionation approaches prior to MS. Despite the recent advancement in gel-free MS techniques, there are some inherent shortcomings that come with this method. MudPIT experiments can be relatively lengthy processes due to the number of fractions produced and the time it takes to analyze each fraction by MS using the reverse phase gradient. Duration of the experiments is the major problem to be resolved in gel-free methods.

## Technical fusion

Several studies that specifically compared gel-free and gel-based proteomics strategies emphasized the complementary nature of the two approaches. Nearly 4000 spots can be processed from a single gel with gel-based methods and over 12,000 proteins can be identified in a sample in the advanced gel-free method. Thus, each method separately produces a significant number of proteins. However, would a combination of both methods (technical fusion) allow us to study an even larger number of proteins? A combination of multiple experiments and analyses has produced a great number of protein identifications (Feng et al., 2009), so a technical fusion would definitely result in a greater number of identified proteins. Moreover, it is not just about the number of proteins/spots, the amount of information obtained about each protein is also important e.g., PTMs. Within gel-based proteomics, BAC gel methods should be fused with the 3D separation techniques to produce greater separation resolution and protein recovery after digestion. 3D BAC separation using two buffer systems would probably be a good technique to consider for not only studying the expressed proteins in a system but also PTMs. On the other hand, similar to the subtraction library system used for creating cDNA libraries, there could be a strategy of protein subtraction and pooling from two different methods. For instance, in a sample processed by both gel-based and gel-free methods, the peptides/proteins (*m*/*z*-value) identified in the gel-based methods could be subtracted using exclusion lists from the *in-solution* digested samples during tandem MS. However, one of the main reasons behind the lack of method development in plant proteomics is budget. The issue of low budgets in plant science laboratories and their effect on proteomic research activities has been mentioned in a recent review by Jorrin-Novo et al. (2015). For example, gel-based techniques, which have been traditionally used in plant proteomics, are now lagging behind in terms of proteome coverage. We urge our fellow plant researchers to move on from our traditional approaches and develop novel strategies like 3D BAC gels or other fusions of techniques and merge different proteomic approaches to better capture proteomic information from the cell.

### Conflict of interest statement

The author declares that the research was conducted in the absence of any commercial or financial relationships that could be construed as a potential conflict of interest.
